# On Uterine Hœmorrhage from Placental Presentation

**Published:** 1818-08

**Authors:** T. Purton

**Affiliations:** Member of the Royal College of Surgeons.


*For the London Medical and Physical Journal.*

*On Uterine Hæmorrhage from Placental Presentation;* by T. PURTON, Member of the Royal College of Surgeons.

WHENEVER hæmorrhage occurs, especially in the latter periods of gestation, it is ever attended with more or less danger to the mother and child. It behoves us, therefore, to be prepared with correct views of the nature of the case, with the surest and readiest means of relieving our patient. The case published in your last Journal by Mr. Edwards, of Bath, reminds me of one I had under my care during my residence in London. The hæmorrhage began between the sixth and seventh months, and recurred at intervals for nearly three weeks; though at first trifling, it increased with each succeeding attack. On making an examination, I was convinced that a large portion of the placenta presented. The os uteri but little dilated. On a consultation with my late worthy and esteemed friend, Dr. Clarke, of New Burlington-street, he advised me, should the hæmorrhage return, to deliver my patient as quickly as possible, or the consequences might be fatal. I did so, and the case terminated favourably. The only difficulty I met with was passing my hand through a rigid os uteri. If in similar cases the hæmorrhage increases with each succeeding recurrence, it is highly necessary to ascertain if the placenta presents; and, if so, there *can* be no sufficient reason why we should defer the delivery: delay would be attended with more *certain* danger to our patient, than any efforts used in the delivery *could* produce. To bleed in such cases (a practice to my knowledge not uncommon) appears to me highly improper in slight detachments of the placenta; if our patient be of a plethoric habit, venesection may be used with advantage; in cases, however, of central presentations of the placenta (or where a considerable portion of it is attached over the os uteri,) such treatment must eventually increase the danger. Allow me then to recommend to my professional brethren, whenever hæmorrhage takes place at the *latter* periods of pregnancy, to make an *early* examination, in order to ascertain correctly the presentation; and, if placental, without hesitation, but slowly and cautiously, prepare for delivery. I feel convinced, and no inconsiderable experience has taught me, that every delay will lessen the chances of recovery. In every case which has come to my knowledge, and terminated *fatally*, it has been *solely* the effect of procrastination.

### *On the Contagion of Influenza.*

Many hundred pages of your Journal were taken up with communications on the above subject, from every part of the British Isles,* in consequence of the late celebrated Dr. Beddoes soliciting for information, in a letter addressed to the Editors:† he also promised to give his own opinion by way of commentary. Among a numerous list of correspondents, I was induced to offer a few observations, which you obliged me by publishing:‡ nor am I aware that any notice has been taken since, excepting a renewal of the doctor's promise in a letter to Dr. Bradley, where he says, "that various interruptions prevented him in the first place from forwarding the concluding observations on the influenza; and that, latterly, he expected to be able to obtain from the continent some valuable intelligence as to its course; a part of its history so necessary towards judging of its contagious or non-contagious nature."§

Should any of your correspondents be acquainted with Dr. B.'s sentiments on this very important and still unsettled point in pathology, they may probably also know his reasons for withholding them from the public.

*Alcester; June* 24, 1818.

* See Medical and Physical Journal, vol. 9, 10, and 11.

† ———————, vol. 10, p. 73.

‡ ———————, vol. 11, p. 81.

§ ———————, vol. 12, p. 479.

